# Burden of Soil-Transmitted Helminth Infections in China: Historical Trends (1990–2021) and Future Projections (2035)

**DOI:** 10.3390/pathogens14111114

**Published:** 2025-11-02

**Authors:** Bixian Ni, Yanzheng Zou, Luqiu Tao, Wei Wang

**Affiliations:** 1 National Health Commission Key Laboratory of Parasitic Diseases Prevention and Control, Wuxi 214064, China; nibixian@jipd.com (B.N.); zouyanzheng@jipd.com (Y.Z.); taoluqiu@jipd.com (L.T.); 2 Jiangsu Provincial Key Laboratory on Parasites and Vector Control Technology, Wuxi 214064, China; 3 Jiangsu Institute of Parasitic Diseases, Wuxi 214064, China

**Keywords:** soil-transmitted helminth infections, disease burden, China, disability-adjusted life years, prevalence, trend analysis

## Abstract

Background: Soil-transmitted helminth (STH) infections, including ascariasis, trichuriasis, and hookworm disease, are among the most common neglected tropical diseases (NTDs) globally. This study evaluates the disease burden of STH in China from 1990 to 2021 and projects trends to 2035. Methods: Data from the Global Burden of Disease 2021 database were utilized to analyze the prevalence and disability-adjusted life years (DALYs) of STH infections in China from 1990 to 2021. The estimated annual percentage change (EAPC) was calculated to assess trends over time, and a Bayesian age-period-cohort model was used to project the disease burden up to 2035. Results: From 1990 to 2021, the prevalence and DALYs of STH infections decreased significantly by 85.08% and 98.01% in China, respectively. The age-standardized prevalence rate (ASPR) of STH infections dropped from 34,073.24/10^5^ to 4981.01/10^5^ with an EAPC of −6.62% [95% confidence interval (*CI*): −7.40%, −5.83%], and the age-standardized DALY rate (ASDR) decreased from 1.77/10^5^ to 0.18/10^5^, with an EAPC of −14.05% (95% *CI*: −15.04%, −13.06%). Trichuriasis contributed to 78.85% of the total ASPR for STH, whereas hookworm disease accounted for 51.14% of STH’s ASDR. The highest disease burden due to STH peaked in the 5–9 years age group, with prevalence of 8030.05/10^5^ [95% uncertainty interval (*UI*): 5356.86/10^5^–11,662.62/10^5^] and DALYs rate of 2.99/10^5^ (95% *UI*: 1.56/10^5^−4.87/10^5^). The projected ASDR and ASPR of trichuriasis rose to 0.55/10^5^ and 5362.50/10^5^ by 2035. Conclusions: China has achieved remarkable reductions in the burden of STH infections over the past three decades. However, the predominance between the species has changed. The projected rebound in trichuriasis underscores the importance of sustained control efforts. To achieve the 2030 elimination target outlined in the WHO NTDs roadmap, it is crucial to integrate precision epidemiology with ongoing water, sanitation, and hygiene initiatives, targeted chemotherapy and health education.

## 1. Introduction

Soil-transmitted helminths (STH), including *Ascaris lumbricoides*, *Trichuris trichiura* and hookworms, require a developmental soil phase for maturation and are transmitted via soil contaminated by human feces [1]. Upon infection, the larvae of *A. lumbricoides* penetrate the gut, migrate via liver and lungs, and then return to the small intestine as adults [2]. *T. trichiura* larvae develop inside gut epithelial cells, and the adults embed in the large intestine [2]. Hookworms, after percutaneous entry and a pulmonary transit, anchor their buccal capsules to the jejunal mucosa [3]. Symptoms of STH infections, such as asthenia, abdominal pain, diarrhea and loss of appetite, are usually non-specific and only noticeable in severe cases. These infections contribute to malnutrition and anemia, leading to adverse effects on physical growth and cognitive development, notably among children [4,5].

STH infections have been recognized as the most common neglected tropical disease (NTDs) by World Health Organization (WHO) [6]. Worldwide, STH infections have impacted around 1.5 billion people, which accounts for 24% of the global population. Predominantly, these infections target impoverished communities in tropical and subtropical regions [7].

In China, STH infections were once a widespread and serious public health concern. With the socioeconomic development and changes in lifestyle, the transmission trends and patterns of STH are undergoing significant changes. The STH has been demonstrated decreased consistently according to the three national surveys of important human parasitic diseases [8,9]. From 1988–1992 to 2014–2015, the overall prevalence rate fell sharply from 53.6% to 4.5% [8,9]. In particular, the prevalence of ascarias dropped from 47.0% to 1.4%, trichuriasis from 18.8% to 1.0%, and hookworm disease from 17.2% to 2.6% [8,9]. Nevertheless, the disease burden in terms of disability-adjusted life years (DALYs) was not assessed in these surveys, and no national epidemiological data has been available since 2016 due to the ongoing fourth national survey. Nowadays, the WHO 2021–2030 road map for NTDs aims to eliminate STH as a public health problem in 96% of countries by 2030 [10].

Consequently, a comprehensive analysis of the epidemiological features of these infections across China is urgently needed to provide a basis for developing targeted prevention and control strategies for STH infections. The Global Burden of Disease Study (GBD) data have offered valuable health measurement across a broad spectrum of health outcomes at global, regional, and national levels [11,12]. This study leveraged the GBD 2021 dataset to evaluate the disease burden of STH infections, encompassing ascariasis, trichuriasis and hookworm disease, in China from 1990 to 2021. It elucidated the prevalence and DALYs across different age groups and genders, and projected the burden of STH through 2035.

## 2. Methods

### 2.1. Data Source

The data utilized in this study were derived from the GBD 2021 open database, which encompasses 204 countries and territories globally and features epidemiological indicators for 371 diseases and injuries [11]. The prevalence and DALYs of STH infections, including ascariasis, trichuriasis, and hookworm disease, were extracted for the period of 1990–2021 using the GBD Results Tool (http://ghdx.healthdata.org/gbd-results-tool) (25 October 2025) available on the Global Health Data Exchange (GHDx) online platform. Cases of STH infections, hookworm disease, ascariasis, and trichuriasis were represented by the International Classification of Diseases (ICD) 10th editions codes: B76-B77, B79.

DALYs were derived by summing years of life lost (YLLs) and years lived with disability (YLDs) of these diseases. The age-standardized prevalence rate (ASPR) and age-standardized DALYs rate (ASDR) were standardized based on the global population from GBD 2021.

### 2.2. Statistical Analysis

The estimated annual percentage change (EAPC) was employed to track trends in both age-standardized and age-specific prevalence and DALY rates of STH infections from 1990 to 2021. The EAPC is calculated by fitting the natural logarithm of the rates to a linear regression model with time as the variable, and then using the slope of this line. The calculation formula was *y* = *α* + *β*x + *ε*, EAPC = 100 × (exp (*β*) − 1). In the model, *y* is the natural logarithm of rates, x is the year, *α* is the intercept, *β* is the slope representing the annual change rate, and *ε* is the error term.

An EAPC of greater than zero indicates an increasing trend, whereas an EAPC of less than zero signifies a decreasing trend. The 95% confidence interval (*CI*) for *β* is computed to evaluate estimate uncertainty. A trend is considered statistically significant if the lower bound of the 95% *CI* is above zero (for an increase) or the upper bound is below zero (for a decrease) [13].

A Bayesian age-period-cohort (BAPC) model was utilized to project the future trends of the disease burden due to STH infections extending to 2030 [14]. Prior investigations have demonstrated that the BAPC model exhibited superior coverage and precision [15,16]. The projected population data from 1990 to 2035 were sourced from the Department of Economic and Social Affairs Population Division of United Nations (https://population.un.org/wpp/) (16 April 2025)

Statistical analyses were conducted using the package R version 4.4.2 (Lucent Technologies, Inc.; Murray Hill, NJ, USA). Projection was conducted using the BAPC package 0.0.36, while data visualization was created by ggplot2 package version 4.0.0, with the significance threshold set at *α* = 0.05.

## 3. Results

### 3.1. Temporal Trends of Disease Burden Due to STH Infections from 1990 to 2021

**Figure 1:**
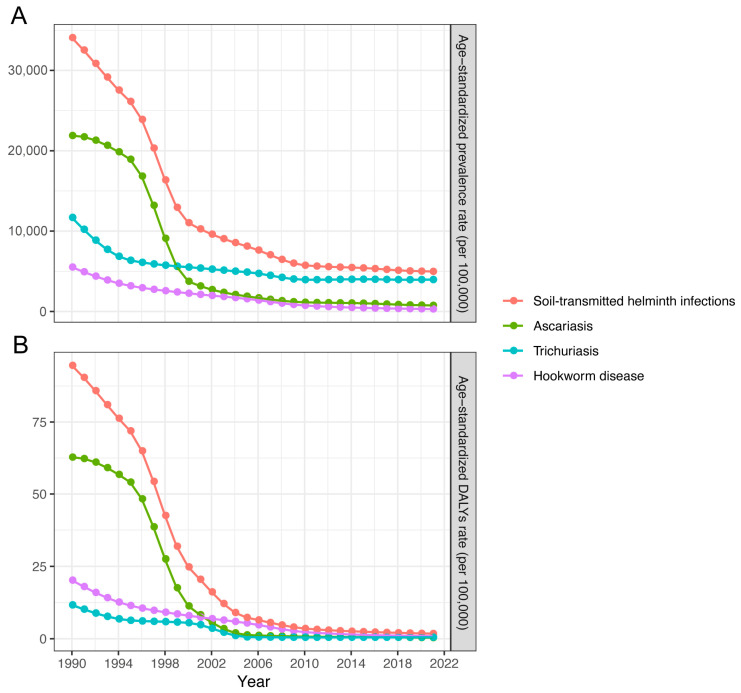
Age-standardized prevalence and DALYs rates of soil-transmitted helminth infections in China from 1990 to 2021. (**A**) Age-standardized prevalence; (**B**) Age-standardized DALYs rate.

**Figure 2:**
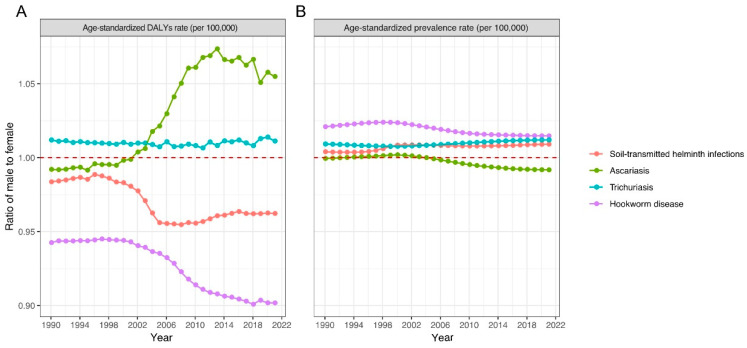
Gender-specific age-standardized DALYs and prevalence rates of soil-transmitted helminth infections in China from 1990 to 2021. (**A**) Age-standardized DALYs rate; (**B**) Age-standardized prevalence.

From 1990 to 2021, the number of prevalent cases of STH infections in China decreased significantly by 85.08%, from 411,914,391.13 (95% *UI*: 322,607,262.30–514,156,693.53) to 61,446,507.17 (95% *UI*: 40,508,264.84–89,273,607.63). Over the same period, the ASPR dropped from 34,073.24/10^5^ (95% *UI*: 26,662.68/10^5^–42,442.11/10^5^) to 4981.01/10^5^ (95% *UI*: 3271.48/10^5^–7261.58/10^5^), with an EAPC of −6.62% (95% *CI*: −7.40%, −5.83%) (Table 1). Throughout this period, a sustained downward trend of ASPR was presented (Figure 1), and the ASPR for STH infections was continuously greater among males than among females (Figure 2).

In 2021, China’s estimated DALYs due to STH infections were 22,657.60 (95% *UI*: 11,600.37–37,867.68), with an ASDR of 1.77/10^5^ (95% *UI*: 0.92/10^5^–2.94/10^5^). From 1990 to 2021, DALYs decreased by 98.01%, with an EAPC of −14.05% (95% *CI*: −15.04%, −13.06%). Similar to the ASPR trend, this period saw a significant decline of ASDR. From 1990 to 2021, females had a higher ASDR compared to males.

### 3.2. Disease Burden Among Specific Species of STH Infections from 1990 to 2021

In terms of parasite species, the largest decline in ASPR was observed for ascariasis, from 21,891.20/10^5^ (95% *UI*: 13,750.91/10^5^–31,616.95/10^5^) to 757.57/10^5^ (95% *UI*: 340.82/10^5^–1451.22/10^5^), with an EAPC of −11.77% (95% *CI*: −13.11%, −10.40%). Meanwhile, the EAPC of trichuriasis and hookworm disease were −2.82% (95% *CI*: −3.29%, −2.35%) and −9.26% (95% *CI*: −9.58%, −8.93%) from 1990 to 2021, respectively (Supplementary Table S1).

The ASDR mirrored the trend of the ASPR, with ascariasis experiencing the most significant decline, having an EAPC of −18.22% (95% *CI*: −20.02%, −16.36%). Over the period from 1990 to 2021, the EAPC was −11.66% (95% *CI*: −13.33%, −9.96%) for trichuriasis, and was −10.02% (95% *CI*: −10.40%, −9.63%) for hookworm disease.

**Figure 3:**
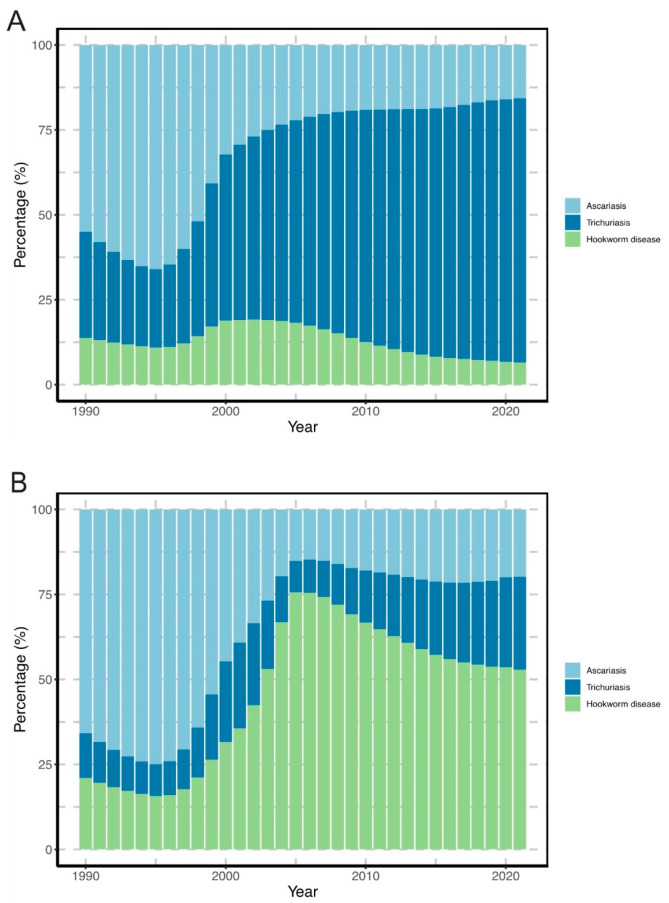
Contribution of ascariasis, trichiasis and hookworm disease to the gross age-standardized prevalence and DALYs rates of soil-transmitted helminth infections in China from 1990 to 2021. (**A**) Age-standardized prevalence rate; (**B**) Age-standardized DALYs rate.

From 1990 to 2021, the proportion of trichuriasis in the ASPR of STH infections rose from 29.91% to 78.85%. In contrast, the proportion of ascariasis dropped from 55.98% to 15.06%, while that of hookworm disease decreased from 14.12% to 6.10%. Regarding ASDR, the proportion of hookworm disease increased from 21.36% to 51.14%, trichuriasis rose slightly from 12.31% to 27.84%, while ascariasis decreased significantly from 66.31% to 21.02% during this period (Figure 3).

In terms of gender, males had a higher ASPR for trichuriasis and hookworm disease during the period. In addition, the ASPR for ascariasis was higher among females than among males from 1990 to 1992. This trend reversed from 1993 to 2004, with a higher ASPR among males. Since 2005, the ASPR among females exceeded that among males once more.

ASDR for hookworm disease was consistently higher among females than among males, while the situation was reversed for trichuriasis. For ascariasis, females had a higher ASDR than males during the period from 1990 to 2001. This pattern changed since 2002, with males having a higher ASDR than females (Figure 2).

### 3.3. Age Patterns of Disease Burden Due to STH Infections from 1990 to 2021

**Figure 4:**
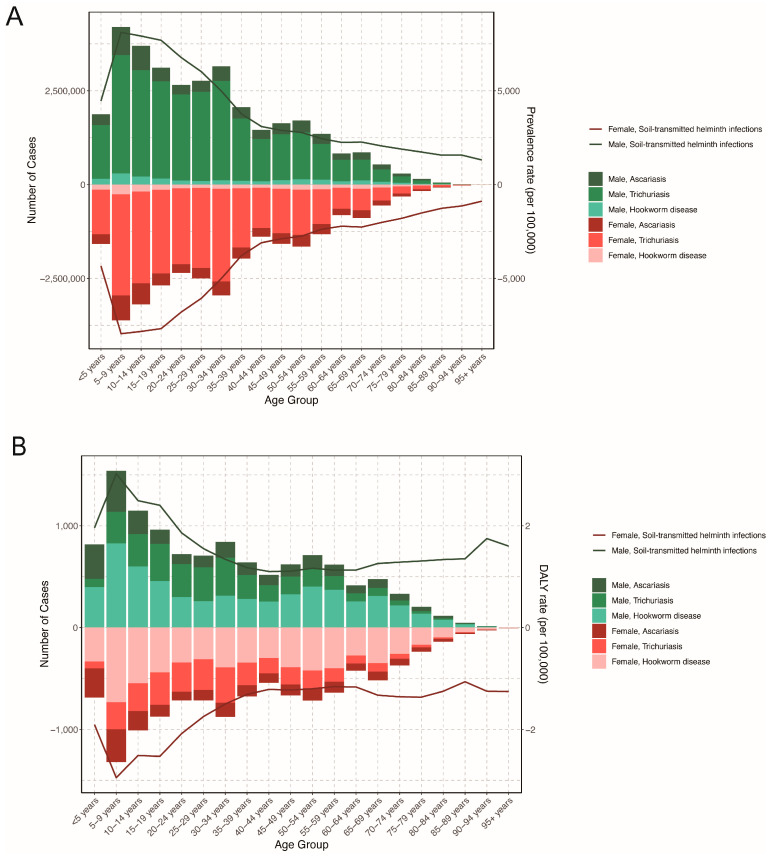
Prevalent cases and DALYs counts of soil-transmitted helminth infections by age groups in China from 1990 to 2021. (**A**) Prevalent cases and prevalence rate; (**B**) DALYs counts and rate.

In 2021, the most prevalent number of STH infections was recorded among children aged 5–9 years [7,690,412.32 (95% *UI*: 5,130,281.22–11,169,338.98)], showing a downward trend with advancing age. The prevalence rate exhibited a similar trend, peaking at 8030.05/10^5^ (95% *UI*: 5356.86/10^5^–11,662.62/10^5^) in this age group (Figure 4).

**Figure 5:**
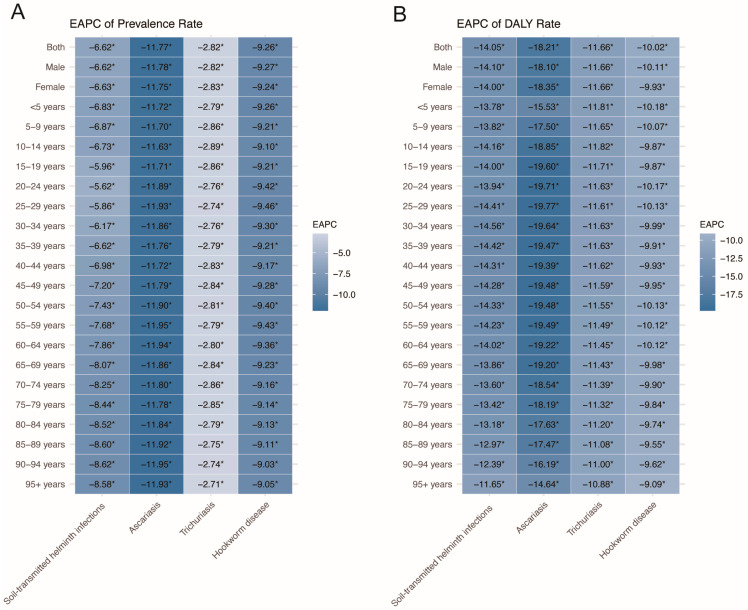
Trends in the prevalence and DALYs rates of soil-transmitted helminth infections by age groups in China from 1990 to 2021. (**A**) Prevalence rate; (**B**) DALYs rate. * Significant difference.

From 1990 to 2021, the prevalent cases of STH infections decreased in most age groups, except for a 29.1% increase among those aged 95 years and above. Meanwhile, the prevalence rate declined across all age groups, with the 90–94 years age group showing the largest decrease [EAPC: −8.62%, 95% *CI*: (−9.49%, −7.75%)] (Figure 5, Supplementary Table S2).

The DALY numbers of STH infections peaked in the 5–9 years age group at 2860.52 cases (95% *UI*: 1495.79–4665.90), declining gradually with age in 2021. From 1990 to 2021, DALY numbers declined across all age groups, with the most substantial reduction in the 20–24 years age group, which saw a 98.86% decrease.

In 2021, the 5–9 years age group had the highest burden of STH infections, with 2.99 (95% *UI*: 1.56–4.87) DALYs per 100,000 people. From 1990 to 2021, DALY rates declined across all age groups, with the most significant reduction in the 30–34 years age group, which had an EAPC of −14.56% [95% *CI*: (−15.78%, −13.32%)] (Supplementary Table S2).

In terms of parasite species, the 5–9 years age group had the highest prevalence rate for *A*. *lumbricoides*, *T*. *trichiura*, and hookworm infections. Similarly, the DALYs for *A*. *lumbricoides* and hookworm infections peaked in the 5–9 years age group. In contrast, the highest DALYs for *T*. *trichiura* infections was observed in the 10–14 years age group in 2021.

From 1990 to 2021, trichuriasis saw the largest declines in prevalence [EAPC: −2.89% (95% *CI*: −3.35%, −2.41%)] and DALYs [EAPC: −11.82% (95% *CI*: −13.51%, −10.08%)] among the 10–14 years age group. Ascariasis had the greatest prevalence decline in both 55–59 years and 90–94 years age groups [EAPC: −11.95% (95% *CI:* −13.32%, −10.56%)] and the largest DALY decline in the 20–24 years age group [EAPC: −19.76% (95% *CI*: −22.05%, −17.41%)]. Hookworm’s highest prevalence decline was seen in the 25–29 years age group [EAPC: −9.46% (95% *CI*: −9.79%, −9.12%)], with the largest DALYs decline in the under 5 years age group [EAPC: −10.18% (95% *CI*: −10.58%, −9.78%)] (Figure 5).

### 3.4. Projections of the Disease Burden Due toSTH Infections up to 2035

**Figure 6:**
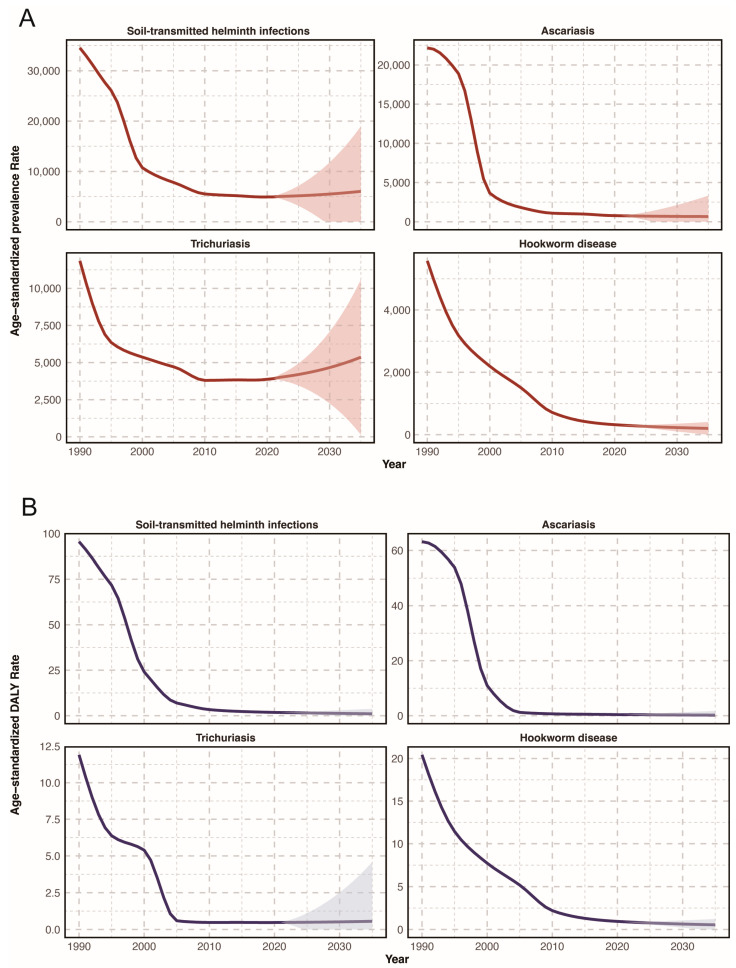
Projections of the prevalence and DALYs rates of soil-transmitted helminth infections in China up to 2035. (**A**) Prevalence rate; (**B**) DALYs rate.

The ASDR of STH infections was projected to decline slightly to 1.07/10^5^ by 2035, indicating a 39.20% reduction from 2021 levels. Conversely, the ASPR was anticipated to rise to 6032.44/10^5^ by 2035 with a 22.23% increase from 2021 (Figure 6).

Regarding specific species, both ASDR and ASPR of *A. lumbricoides* and hookworm infections were predicted to continue to decline to 2035. However, the ASDR and ASPR for *T. trichiura* were both projected to experience an increase to 0.55/10^5^ and 5362.50/10^5^, with a 16.47% and 49.97% rise from 2021 (Supplementary Table S3).

## 4. Discussion

This study offers a comprehensive analysis of the spatial and temporal dynamics of STH infections in China across a 30-year span, with projections extending to 2035. We demonstrate a sustained, marked decline in both prevalence (85.08%) and DALYs (98.01%) between 1990 and 2021, while uncovering varied trends across age, gender and parasite species.

The pronounced decline in the disease burden of STH infections in China from 1990 to 2021 was attributed to sustained socioeconomic development, poverty reduction, improved water, sanitation, and hygiene (WASH) access, and periodic deworming campaigns [17]. In 1992, the China’s national parasitic disease control program (“eighth five-year” plan and 2000 plan) was issued, and mass deworming campaigns were launched [18]. Consequently, the ASPR and ASDR of STH infections experienced the most significant declines during the period from 1992 to 2000. The steeper EAPC for ASDR (−14.05%) relative to ASPR (−6.62%) indicates that not only infection frequency but also clinical severity and sequelae have diminished [19], consistent with observations that periodic anthelmintic treatment can reduce stunting and anemia even when transmission continues [20].

Not only has the burden of STH decreased markedly, but the predominance between the special species has also changed. Trichuriasis accounted for 78.85% of the ASPR and hookworm disease for 51.14% of the ASDR in 2021, reversing the historical dominance of ascariasis observed in the 1988–1992 national survey [6]. Improved sanitation is associated with a lower risk of trichuriasis and ascariasis, but not with hookworm disease [21]. Previous studies have identified barefoot farming as a significant risk factor for hookworm infection, with a higher barefoot labor frequency correlating to an increased infection risk [22,23]. Thus, as the dominant species of STH, implementation of community-based intervention such as altering the practice of barefoot labor is crucial for controlling hookworm disease [24].

The prevalence and DALYs of STH infection peaked in the 5–9 years age group and declined with increasing age, aligning with findings from the STH’s global burden study [25] and other studies [26,27]. This also supports the WHO’s assertion that school-aged children remain the sentinel population for STH transmission. Factors such as the number of siblings, maternal education level and drinking unboiled water were found to correlate with the risk of STH infections among school-aged children [28]. The burden of STH declined in all other age groups, yet the number of prevalent cases rose by 29.1% among those aged over 95 years, likely attributable to the population ageing in China [29]. The elderly may be more susceptible to STH infections due to the decline of immunity.

In terms of gender, males consistently exhibited a higher ASPR of STH infections than females, whereas females consistently had a higher ASDR than males throughout the study period. This finding aligns with a previous systematic analysis of the STH burden in China from the GBD 2019 study [30]. Men’s higher ASPR may stem from their frequent agricultural work. In some rural areas, men’s higher social status grants them better healthcare access, leading to higher diagnosis rates of STH infections. In contrast, women, often undiagnosed, likely bear a higher disease burden [31].

By 2035, the ASDR of STH infections was projected to decline, while the ASPR was expected to increase slightly. Notably, trichuriasis was anticipated to experience a significant rebound, with its ASDR and ASPR were projected to increase by 16.47% and 49.97%, respectively. This highlights the potential resurgence of the disease if intervention coverage wanes, alerting Chinese policymakers to the risk of recrudescence without sustained control programmes. Benzimidazoles are less effective against *T. trichiura* infections, with a single-dose cure rate of around 30%, compared to 90–95% for *A. lumbricoides* infections [32]. Additionally, there is a possibility that drug resistance may become prevalent, which could derail efforts to control *T. Trichiura* transmission [33]. Monitoring benzimidazole efficacy and exploring new treatment strategies are essential for controlling trichuriasis [34]. Furthermore, genetic analyses have highlighted cross-host species infections of *A. lumbricoides* and *T. trichiura* between humans and pigs [35,36]. These findings underscore the potential for zoonotic transmission and support the necessity of a One Health approach to control the spread of human STH infections.

This study also has several limitations. Firstly, it relies on the GBD database, which aggregates data only at the national level and does not include province-level data of China. This may obscure important epidemiological nuances across different regions. Secondly, the GBD 2021 database covers only three types of STH—*A. lumbricoides*, *T. trichiura* and hookworm, and excludes other significant species such as *Strongyloides stercoralis* and *Enterobius vermicularis*. This exclusion limits the comprehensive understanding of the prevalence of STH infections. The true prevalence of these infections remains unclear, highlighting the need for further systematic parasitological surveys to accurately assess their impact on human health.

## 5. Conclusions

Over the past three decades, China has achieved remarkable reductions in STH infections. School-aged children have borne the highest burden of STH infections. The disease burden attributed to trichuriasis and hookworm infections surpasses that of ascariasis. Projections suggest that prevalence and DALYs of trichuriasis may increase by 2035. To achieve the 2030 elimination target outlined in the WHO NTDs roadmap, it will be crucial to integrate precision epidemiology with ongoing WASH initiatives, targeted chemotherapy and health education.

## Figures and Tables

**Table 1:** Trends in prevalence and DALYs of STH infections in China from 1990 to 2021.

Gender	Prevalent Cases (95% *UI*)		Percentage Change (%)	ASPR (10^5^, 95% *UI*)		EAPC (%, 95% *CI*)	DALYs Counts (95%*UI*)		Percentage Change (%)	ASDR (10^5^, 95% *UI*)		EAPC (%, 95% *CI*)
	1990	2021		1990	2021		1990	2021		1990	2021	
Both	411,914,391.13 (322,607,262.30–514,156,693.53)	61,446,507.17 (40,508,264.84–89,273,607.63)	−85.08	34,073.24 (26,662.68–42,442.11)	4981.01 (3271.48–7261.58)	−6.62 (−7.40, −5.83)	1,138,511.95 (611,146.80–1,835,589.65)	22,657.60 (11,600.37–37,867.68)	−98.01	94.66 (51.04, 152.49)	1.77 (0.92, 2.94)	−14.05 (−15.04, −13.06)
Male	212,759,238.92 (166,746,039.35–265,554,106.75)	32,109,355.75 (21,155,114.45–46,672,781.25)	−84.91	34,153.27 (26,745.66–42,517.89)	5003.27 (3285.67–7295.82)	−6.62 (−7.40, −5.83)	581,546.75 (311,609.15–939,508.82)	11,450.05 (5865.84–18,973.53)	−98.03	93.96 (50.53, 151.71)	1.74 (0.90, 2.86)	−14.10 (−15.10, −13.09)
Female	199,155,152.21 (155,861,222.94–248,602,586.79)	29,337,151.42 (19,372,543.23–42,628,170.24)	−85.27	34,016.90 (26,596.79–42,398.10)	4958.14 (3257.02–7226.14)	−6.63 (−7.42, −5.84)	556,965.20 (299,776.15–896,253.92)	11,207.55 (5731.94–18,764.57)	−97.99	95.52 (51.70, 153.50)	1.81 (0.94, 3.00)	−14.00 (−14.98, −13.02)

Abbreviations: ASPR, age-standardized prevalence rate; DALYs, disability adjusted life years; ASDR, age-standardized DALY rate; EAPC, estimated annual percentage change; *UI*, uncertainty interval; *CI*, confidence interval.

## Data Availability

All data presented in this study are available upon request by contact with the corresponding author.

## Supplementary Materials

The following supporting information can be downloaded at: https://www.mdpi.com/article/10.3390/pathogens14111114/s1. Table S1 Prevalence and DALYs trends of specific species of STH in China from 1990 to 2021. Table S2 Age-specific prevalence of STH infections in China in 1990 and 2021. Table S3 Projection of DALYs for STH infections in China up to 2035.
