# Supplementary material for: Burden of Soil-Transmitted Helminth Infections in China: Historical Trends (1990–2021) and Future Projections (2035)
Source: Pathogens. 2025 Nov 2;14(11):1114. doi: 10.3390/pathogens14111114 (PMC12655046; doi:10.3390/pathogens14111114)
Supplement: Supplementary file 1 [file pathogens-14-01114-s001.zip › pathogens-3941073-supplementary.pdf]

Table S1 Prevalence and DALYs trends of specific species of STH in China from 1990 to 2021

Species	Gender	Prevalent cases, 95% UI		Percentage change (%)	ASPR (per 100 000), 95% UI		EAPC (%, 95% CI)	DALYs cases, 95%UI		Percentage change (%)	ASDR (per 100 000), 95% UI		EAPC (%, 95% CI)
		1990	2021		1990	2021		1990	2021		1990	2021	
Ascariasis	Both	260,530,454.77 (163,790,380.84, 376,128,127.58)	9,724,352.35 (4,373,858.16, 18,626,851.86)	-96.27	21,891.20 (13,750.91, 31,616.95)	757.57 (340.82, 1,451.22)	-11.77 (-13.11, -10.40)	749,655.17 (383,671.38, 1,247,020.95)	4,493.47 (2,287.36, 8,442.32)	-99.4	62.77 (32.30, 104.19)	0.37 (0.20, 0.68)	-18.22 (-20.02, -16.36)
	Male	134,318,175.74 (84,459,159.09, 193,869,876.94)	5,016,528.74 (2,255,619.83, 9,609,055.79)	-96.27	21,896.20 (13,757.13, 31,616.48)	754.85 (339.71, 1,446.15)	-11.78 (-13.12, -10.42)	385,738.84 (197,392.58, 644,267.95)	2,391.68 (1,230.92, 4,460.31)	-99.38	62.57 (32.15, 104.38)	0.38 (0.21, 0.69)	-18.09 (-19.90, -16.25)
	Female	126,212,279.03 (79,331,221.75, 182,258,250.64)	4,707,823.61 (2,120,174.02, 9,017,796.08)	-96.27	21,907.73 (13,762.55, 31,648.87)	761.13 (342.77, 1,457.87)	-11.75 (-13.09, -10.38)	363,916.33 (186,375.40, 602,753.00)	2,101.79 (1,043.42, 3,981.23)	-99.42	63.07 (32.55, 104.11)	0.36 (0.19, 0.67)	-18.35 (-20.16, -16.49)

Trichuriasis	Both	147,772,098.22 (96,165,240.02, 218,150,775.51)	48,272,112.85 (27,731,899.09, 75,357,962.83)	-67.33	11,695.06 (7,615.58, 17,261.62)	3,966.50 (2,275.59, 6,197.75)	-2.82 (-3.29, -2.35)	749,655.17 (383,671.38, 1,247,020.95)	4,493.47 (2,287.36, 8,442.32)	-99.4	11.66 (5.49, 21.64)	0.49 (0.20, 0.97)	-11.66 (-13.33, -9.96)
	Male	76,577,921.45 (49,813,064.09, 113,014,379.77)	25,300,121.62 (14,523,653.00, 39,493,836.01)	-66.96	11,748.28 (7,646.95, 17,335.48)	3,989.31 (2,287.74, 6,230.89)	-2.82 (-3.29, -2.34)	385,738.84 (197,392.58, 644,267.95)	2,391.68 (1,230.92, 4,460.31)	-99.38	11.73 (5.55, 21.82)	0.49 (0.20, 0.97)	-11.67 (-13.34, -9.97)
	Female	71,194,176.77 (46,349,674.02, 105,136,395.73)	22,971,991.23 (13,208,246.09, 35,864,126.82)	-67.73	11,640.58 (7,583.18, 17,186.03)	3,941.99 (2,262.50, 6,159.69)	-2.83 (-3.30, -2.36)	363,916.33 (186,375.40, 602,753.00)	2,101.79 (1,043.42, 3,981.23)	-99.42	11.59 (5.43, 21.46)	0.49 (0.19, 0.97)	-11.66 (-13.33, -9.94)
Hookworm disease	Both	65,228,457.58 (42,122,088.24, 96,722,690.08)	4,006,012.54 (2,530,257.00, 5,971,398.15)	-93.86	5,521.07 (3,566.06, 8,191.32)	306.63 (193.64, 457.20)	-9.26 (-9.58, -8.93)	238,783.89 (117,009.23, 436,471.44)	11,978.24 (5,512.81, 20,516.65)	-94.98	20.22 (9.93, 36.91)	0.90 (0.42, 1.56)	-10.02 (-10.40, -9.63)

Male	33,867,725.26 (21,870,296.77, 50,214,576.25)	2,085,829.84 (1,317,354.91, 3,109,146.80)	-93.84	5,580.16 (3,604.09, 8,279.12)	309.00 (195.11, 460.75)	-9.27 (-9.60, -8.95)	118,030.65 (57,146.17, 215,647.51)	5,823.52 (2,633.77, 10,078.62)	-95.07	19.65 (9.56, 35.89)	0.86 (0.39, 1.50)	-10.10 (-10.49, -9.71)
Female	31,360,732.32 (20,251,791.46, 46,508,113.83)	1,920,182.70 (1,212,902.09, 2,862,251.35)	-93.88	5,465.60 (3,530.26, 8,109.51)	304.51 (192.33, 454.02)	-9.24 (-9.56, -8.92)	120,753.23 (59,863.06, 220,432.98)	6,154.72 (2,855.79, 10,502.33)	-94.9	20.85 (10.35, 38.02)	0.95 (0.45, 1.64)	-9.93 (-10.31, -9.56)

Abbreviations: ASPR, age-standardized prevalence rate; DALYs, disability adjusted life years; ASDR, age-standardized DALY rate; EAPC, estimated annual percentage change; UI, uncertainty interval; CI, confidence interval

Table S2 Age-specific prevalence of STH in China in 1990 and 2021

Age group (years)	Prevalent cases, 95% UI		Percentage change (%)	ASPR (per 100 000), 95% UI		EAPC (%, 95% CI)	DALYs cases, 95%UI		Percentage change (%)	ASDR (per 100 000), 95% UI		EAPC (%, 95% CI)
	1990	2021		1990	2021		1990	2021		1990	2021	
＜5	32,947,784.89 (25,887,037.71, 40808685.10)	3,416,081.74 (2,262,781.13, 4,947,110.22)	-89.63	29,468.70 (23,153.53, 36,499.54)	4,398.30 (2,913.39, 6,369.54)	-6.83 (-7.63, -6.04)	119,917.61 (83,798.82, 164,813.60)	1,501.30 (919.70, 2,304.73)	-98.75	107.26 (74.95, 147.41)	1.93 (1.18, 2.97)	-13.78 (-14.50, -13.06)
5-9	59,317,962.33 (46,996,491.51, 73,920,381.00)	7,690,412.32 (5,130,281.22, 11,169,338.98)	-87.04	56,884.48 (45,068.49, 70,887.84)	8,030.05 (5,356.86, 11,662.62)	-6.87 (-7.63, -6.09)	159,447.45 (85,306.04, 260,286.23)	2,860.52 (1,495.79, 4,665.90)	-98.21	152.91 (81.81, 249.61)	2.99 (1.56, 4.87)	-13.82 (-14.70, -12.94)
10-14	56,084,575.01 (44,405,029.65, 69,798,087.15)	6,785,730.50 (4,489,099.46, 9,880,744.68)	-87.9	54,827.70 (43,409.89, 68,233.88)	7,872.72 (5,208.20, 11,463.52)	-6.73 (-7.53, -5.93)	141,778.69 (71,974.64, 234,550.95)	2,155.95 (1,069.35, 3,644.38)	-98.48	138.60 (70.36, 229.29)	2.50 (1.24, 4.23)	-14.16 (-15.16, -13.14)
15-19	56,574,525.87 (44,862,403.32, 69,080,492.91)	5,736,368.42 (3,717,093.61, 8,533,438.77)	-89.86	44,664.61 (35,418.10, 54,537.86)	7,682.07 (4,977.88, 11,427.87)	-5.96 (-6.68, -5.23)	159,384.89 (80,939.79, 259,652.48)	1,836.92 (913.21, 3,083.19)	-98.85	125.83 (63.90, 204.99)	2.46 (1.22, 4.13)	-14.00 (-15.09, -12.89)
20-24	47,908,683.47 (37,314,905.42, 58,592,182.72)	4,961,367.16 (3,135,494.88, 7,419,755.06)	-89.64	36,294.21 (28,268.67, 44,387.71)	6,780.22 (4,284.98, 10,139.86)	-5.62 (-6.34, -4.91)	125,907.27 (65,139.82, 205,015.76)	1,435.72 (715.25, 2,462.41)	-98.86	95.38 (49.35, 155.31)	1.96 (0.98, 3.37)	-13.94 (-15.09, -12.78)
25-29	37,286,800.47 (28,567,518.16, 46,241,032.11)	5,223,774.01 (3,300,168.25, 7,807,348.34)	-85.99	33,931.19 (25,996.60, 42,079.59)	6,040.30 (3,816.01, 9,027.71)	-5.86 (-6.62, -5.10)	96,624.82 (49,468.73, 158,230.48)	1,420.70 (704.15, 2,458.53)	-98.53	87.93 (45.02, 143.99)	1.64 (0.81, 2.84)	-14.41 (-15.66, -13.15)

30-34	26,967,790.31 (20,550,049.22, 34,160,530.75)	6,057,213.32 (3,857,670.99, 8,985,011.29)	-77.54	30,560.37 (23,287.67, 38,711.31)	4,999.63 (3,184.12, 7,416.23)	-6.17 (-6.96, -5.37)	70,419.00 (35,359.40, 115,797.22)	1,716.05 (852.91, 2,901.39)	-97.56	79.80 (40.07, 131.22)	1.42 (0.70, 2.39)	-14.56 (-15.78, -13.32)
35-39	24,038,220.73 (18,357,588.60, 30,569,441.72)	4,005,285.65 (2,604,850.79, 5,901,287.54)	-83.34	26,317.57 (20,098.29, 33,468.09)	3,779.88 (2,458.26, 5,569.18)	-6.62 (-7.45, -5.79)	64,990.80 (32,820.66, 106,487.50)	1,315.13 (664.33, 2,209.88)	-97.98	71.15 (35.93, 116.58)	1.24 (0.63, 2.09)	-14.42 (-15.53, -13.29)
40-44	15,933,626.14 (12,233,820.52, 20,219,005.44)	2,834,616.63 (1,880,621.57, 4,108,266.40)	-82.21	23,748.09 (18,233.76, 30,135.19)	3,096.80 (2,054.57, 4,488.26)	-6.98 (-7.84, -6.12)	44,058.99 (22,399.24, 72,918.38)	1,057.15 (526.72, 1,796.16)	-97.6	65.67 (33.38, 108.68)	1.15 (0.58, 1.96)	-14.31 (-15.39, -13.21)
45-49	12,155,969.29 (9,378,594.02, 15,437,155.54)	3,186,892.39 (2,140,618.22, 4,621,443.34)	-73.78	23,549.43 (18,168.90, 29,905.98)	2,888.73 (1,940.34, 4,189.07)	-7.20 (-8.06, -6.33)	33,812.96 (17,142.30, 55,351.61)	1,284.55 (633.00, 2,202.49)	-96.2	65.50 (33.21, 107.23)	1.16 (0.57, 2.00)	-14.28 (-15.37, -13.17)
50-54	11,294,470.27 (8,726,313.93, 14,419,219.06)	3,339,146.99 (2,253,376.78, 4,834,651.19)	-70.44	23,672.74 (18,289.99, 30,222.08)	2,762.84 (1,864.47, 4,000.24)	-7.43 (-8.29, -6.56)	31,614.30 (15,959.95, 51,953.07)	1,428.19 (703.20, 2,405.08)	-95.48	66.26 (33.45, 108.89)	1.18 (0.58, 1.99)	-14.34 (-15.41, -13.26)
55-59	9,544,882.11 (7,329,295.91, 12,280,343.30)	2,654,656.21 (1,809,279.11, 3,823,541.89)	-72.19	22,008.48 (16,899.81, 28,315.88)	2,414.59 (1,645.66, 3,477.77)	-7.68 (-8.52, -6.83)	26,870.94 (13,530.19, 44,086.82)	1,255.16 (643.27, 2,136.72)	-95.33	61.96 (31.20, 101.65)	1.14 (0.59, 1.94)	-14.23 (-15.23, -13.23)
60-64	7,596,678.90 (5,757,334.37, 9,774,479.44)	1,631,102.18 (1,114,022.29, 2,330,926.37)	-78.53	21,497.52 (16,292.44, 27,660.39)	2,234.23 (1,525.95, 3,192.82)	-7.86 (-8.68, -7.04)	21,818.54 (11,106.93, 35,282.95)	837.65 (418.97, 1,403.81)	-96.16	61.74 (31.43, 99.85)	1.15 (0.57, 1.92)	-14.01 (-14.89, -13.12)
65-69	6,363,426.75 (4,798,765.56, 8,174,681.25)	1,737,521.28 (1,198,802.65, 2,468,360.91)	-72.7	23,324.80 (17,589.61, 29,963.85)	2,265.24 (1,562.90, 3,218.05)	-8.07 (-8.89, -7.25)	18,547.84 (9,543.06, 29,737.33)	991.09 (485.88, 1,646.11)	-94.66	67.99 (34.98, 109.00)	1.29 (0.63, 2.15)	-13.86 (-14.71, -13.01)

70-74	4,183,144.81 (3,148,448.72, 5,374,403.87)	1,085,225.27 (751,985.97, 1,535,171.59)	-74.06	22,229.90 (16,731.36, 28,560.44)	2,036.21 (1,410.95, 2,880.44)	-8.25 (-9.07, -7.41)	12,322.10 (6,471.35, 19,553.55)	704.27 (357.70, 1,192.31)	-94.28	65.48 (34.39, 103.91)	1.32 (0.67, 2.24)	-13.60 (-14.43, -12.76)
75-79	2,400,431.04 (1,800,378.60, 3,094,148.46)	609,112.28 (427,997.04, 860,015.85)	-74.62	21,092.13 (15,819.58, 27,187.69)	1,839.17 (1,292.30, 2,596.75)	-8.44 (-9.28, -7.59)	7,108.55 (3,724.16, 11,350.27)	442.57 (238.60, 727.34)	-93.77	62.46 (32.72, 99.73)	1.34 (0.72, 2.20)	-13.42 (-14.26, -12.58)
80-84	988,566.80 (739,551.64, 1,277,806.04)	318,219.62 (222,343.48, 446,739.38)	-67.81	18,662.33 (13,961.38, 24,122.63)	1,607.83 (1,123.41, 2,257.19)	-8.52 (-9.37, -7.66)	2,915.82 (1,563.02, 4,603.39)	254.53 (140.48, 412.91)	-91.27	55.05 (29.51, 86.90)	1.29 (0.71, 2.09)	-13.17 (-13.99, -12.35)
85-89	275,755.23 (205,843.15, 357,553.58)	130,817.38 (91,586.32, 182,700.41)	-52.56	16,347.29 (12,202.77, 21,196.46)	1,373.30 (961.46, 1,917.96)	-8.60 (-9.44, -7.74)	805.72 (421.26, 1,271.47)	111.08 (60.55, 178.77)	-86.21	47.76 (24.97, 75.38)	1.17 (0.64, 1.88)	-12.97 (-13.76, -12.18)
90-94	46,320.35 (34,608.09, 60,243.21)	36,797.53 (25,739.24, 51,405.81)	-20.56	15,096.81 (11,279.53, 19,634.58)	1,255.04 (877.88, 1,753.28)	-8.62 (-9.49, -7.75)	149.81 (81.28, 232.57)	40.63 (22.97, 63.79)	-72.88	48.83 (26.49, 75.80)	1.39 (0.78, 2.18)	-12.39 (-13.07, -11.70)
>95	4,776.37 (3,547.47, 6,230.30)	6,166.30 (4,274.16, 8,626.06)	29.1	11,795.82 (8,760.90, 15,386.46)	964.84 (668.78, 1,349.72)	-8.58 (-9.48, -7.66)	15.85 (9.27, 24.12)	8.42 (5.09, 12.87)	-46.88	39.15 (22.89, 59.56)	1.32 (0.80, 2.01)	-11.65 (-12.34, -10.96)

Abbreviations: ASPR, age-standardized prevalence rate; DALYs, disability adjusted life years; ASDR, age-standardized DALY rate; EAPC, estimated annual percentage change; UI, uncertainty interval; CI, confidence interval

Table S3 Projection of DALYs for STH in China up to 2035

Year	STH		Ascariasis		Trichuriasis		Hookworm disease	
	Prevalence (per 100,000), 95% CI	DALYs (per 100,000) , 95% CI	Prevalence (per 100,000), 95% CI	DALYs (per 100,000), 95% CI	Prevalence (per 100,000), 95% CI	DALYs (per 100,000), 95% CI	Prevalence (per 100,000), 95% CI	DALYs (per 100,000), 95% CI
1990	34533.56 (34530.13, 34537.01)	95.53 (95.35, 95.71)	22173.88 (22171.11, 22176.65)	63.18 (63.04, 63.33)	11851.91 (11849.95, 11853.86)	11.91 (11.85, 11.97)	5573.31 (5571.91, 5574.70)	20.43 (20.35, 20.52)
1991	32964.12 (32960.78, 32967.45)	91.3 (91.13, 91.48)	22014.33 (22011.60, 22017.07)	62.7 (62.56, 62.85)	10351.76 (10349.94, 10353.57)	10.40 (10.35, 10.46)	4979.92 (4978.61, 4981.23)	18.19 (18.11, 18.27)
1992	31234.66 (31231.43, 31237.88)	86.59 (86.42, 86.76)	21555.43 (21552.75, 21558.12)	61.45 (61.31, 61.59)	8974.1 (8972.41, 8975.78)	9.01 (8.96, 9.06)	4430.34 (4429.12, 4431.57)	16.12 (16.05, 16.20)
1993	29410.43 (29407.33, 29413.53)	81.45 (81.29, 81.62)	20826.06 (20823.44, 20828.68)	59.37 (59.23, 59.51)	7792.61 (7791.05, 7794.17)	7.82 (7.77, 7.87)	3934.34 (3933.20, 3935.49)	14.26 (14.19, 14.32)
1994	27661.01 (27658.02, 27663.99)	76.42 (76.26, 76.58)	19922.31 (19919.77, 19924.85)	56.81 (56.68, 56.95)	6896.08 (6894.62, 6897.53)	6.92 (6.87, 6.97)	3513.34 (3512.27, 3514.42)	12.68 (12.62, 12.74)
1995	26086.62 (26083.73, 26089.50)	71.7 (71.55, 71.85)	18878.23 (18875.78, 18880.69)	53.9 (53.77, 54.03)	6356.81 (6355.42, 6358.21)	6.37 (6.33, 6.42)	3179.15 (3178.13, 3180.17)	11.42 (11.37, 11.48)

1996	23732.63 (23729.89, 23735.37)	64.49 (64.35, 64.64)	16707.84 (16705.53, 16710.14)	47.92 (47.79, 48.04)	6065.09 (6063.73, 6066.45)	6.10 (6.06, 6.15)	2924.65 (2923.68, 2925.63)	10.46 (10.40, 10.51)
1997	20106.79 (20104.27, 20109.30)	53.7 (53.57, 53.83)	13056.84 (13054.81, 13058.87)	38.1 (37.99, 38.21)	5838.68 (5837.34, 5840.01)	5.93 (5.89, 5.97)	2713.59 (2712.66, 2714.53)	9.66 (9.61, 9.72)
1998	16098.87 (16096.63, 16101.11)	41.76 (41.64, 41.87)	8952.59 (8950.91, 8954.27)	26.98 (26.89, 27.07)	5657.7 (5656.39, 5659.01)	5.79 (5.75, 5.83)	2528.63 (2527.73, 2529.54)	8.97 (8.92, 9.02)
1999	12666.20 (12664.22, 12668.19)	31.11 (31.01, 31.21)	5468.47 (5467.16, 5469.78)	17.12 (17.05, 17.20)	5507.11 (5505.82, 5508.40)	5.63 (5.59, 5.67)	2360.45 (2359.58, 2361.33)	8.34 (8.29, 8.39)
2000	10738.95 (10737.12, 10740.78)	24.09 (24.01, 24.18)	3645.74 (3644.67, 3646.81)	10.96 (10.9, 11.01)	5369.72 (5368.44, 5370.99)	5.39 (5.35, 5.43)	2197.28 (2196.43, 2198.12)	7.73 (7.69, 7.78)
2001	9951.73 (9949.97, 9953.49)	19.84 (19.77, 19.92)	3069.66 (3068.68, 3070.64)	7.96 (7.90, 8.01)	5235.47 (5234.21, 5236.74)	4.71 (4.68, 4.75)	2045.68 (2044.87, 2046.50)	7.17 (7.13, 7.22)
2002	9293.42 (9291.71, 9295.12)	15.58 (15.51, 15.65)	2626.53 (2625.62, 2627.45)	5.39 (5.35, 5.43)	5098.2 (5096.95, 5099.46)	3.51 (3.48, 3.55)	1909.04 (1908.25, 1909.83)	6.67 (6.62, 6.71)
2003	8732.97 (8731.36, 8734.63)	11.65 (11.59, 11.71)	2285.64 (2284.79, 2286.50)	3.3 (3.26, 3.33)	4962.86 (4961.62, 4964.09)	2.17 (2.14, 2.19)	1778.04 (1777.27, 1778.80)	6.18 (6.14, 6.22)

2004	8245.62 (8244, 8247.23)	8.63 (8.57, 8.68)	2021.3 (2020.50, 2022.11)	1.86 (1.84, 1.89)	4833.83 (4832.61, 4835.06)	1.07 (1.05, 1.09)	1645.64 (1644.9, 1646.37)	5.69 (5.65, 5.73)
2005	7809.82 (7808.25, 7811.40)	6.97 (6.93, 7.02)	1810.56 (1809.79, 1811.32)	1.21 (1.19, 1.24)	4716.6 (4715.39, 4717.82)	0.59 (0.58, 0.61)	1505.14 (1504.44, 1505.85)	5.17 (5.14, 5.21)
2006	7329.68 (7328.15, 7331.21)	6.15 (6.10, 6.19)	1619.39 (1618.67, 1620.12)	1.04 (1.02, 1.06)	4556.14 (4554.94, 4557.34)	0.55 (0.54, 0.56)	1343.33 (1342.67, 1344)	4.56 (4.53, 4.60)
2007	6770.89 (6769.42, 6772.37)	5.31 (5.26, 5.35)	1437.1 (1436.42, 1437.79)	0.92 (0.9, 0.94)	4326.3 (4325.13, 4327.47)	0.52 (0.51, 0.53)	1162.42 (1161.8, 1163.04)	3.87 (3.84, 3.90)
2008	6219.46 (6218.04, 6220.88)	4.5 (4.47, 4.54)	1278.16 (1277.51, 1278.81)	0.83 (0.81, 0.84)	4083.71 (4082.57, 4084.86)	0.5 (0.49, 0.51)	982.49 (981.92, 983.06)	3.18 (3.15, 3.21)
2009	5768.11 (5766.74, 5769.48)	3.82 (3.79, 3.86)	1157.13 (1156.51, 1157.75)	0.75 (0.73, 0.76)	3888 (3886.89, 3889.12)	0.48 (0.47, 0.49)	825.41 (824.89, 825.94)	2.60 (2.58, 2.63)
2010	5515.91 (5514.57, 5517.25)	3.35 (3.31, 3.38)	1089.26 (1088.65, 1089.86)	0.67 (0.66, 0.69)	3803.1 (3801.99, 3804.21)	0.48 (0.47, 0.49)	713.29 (712.80, 713.78)	2.20 (2.18, 2.23)
2011	5417.35 (5416.02, 5418.68)	3.05 (3.02, 3.08)	1059.95 (1059.35, 1060.55)	0.63 (0.61, 0.64)	3803.83 (3802.71, 3804.94)	0.48 (0.47, 0.49)	637 (636.54, 637.46)	1.94 (1.92, 1.96)
2012	5341.25 (5339.92, 5342.57)	2.81 (2.78, 2.84)	1038.48 (1037.89, 1039.07)	0.59 (0.58, 0.61)	3810.68 (3809.56, 3811.79)	0.48 (0.47, 0.49)	570.52 (570.08, 570.96)	1.73 (1.71, 1.75)

2013	5279.23 (5277.92, 5280.55)	2.6 (2.57, 2.63)	1020.69 (1020.1, 1021.27)	0.56 (0.55, 0.58)	3819 (3817.88, 3820.11)	0.48 (0.47, 0.49)	513.86 (513.45, 514.28)	1.56 (1.54, 1.58)
2014	5227.78 (5226.47, 5229.09)	2.44 (2.42, 2.47)	1003.2 (1002.62, 1003.78)	0.55 (0.53, 0.56)	3827.97 (3826.85, 3829.09)	0.48 (0.47, 0.49)	467.37 (466.98, 467.77)	1.42 (1.40, 1.43)
2015	5177.36 (5176.06, 5178.67)	2.31 (2.28, 2.34)	980.74 (980.16, 981.31)	0.53 (0.52, 0.54)	3834.22 (3833.1, 3835.34)	0.48 (0.47, 0.49)	429.78 (429.40, 430.15)	1.30 (1.28, 1.32)
2016	5109.71 (5108.41, 5111.01)	2.19 (2.16, 2.21)	941.91 (941.35, 942.47)	0.51 (0.5, 0.52)	3831.86 (3830.73, 3832.99)	0.48 (0.47, 0.49)	399.41 (399.04, 399.77)	1.20 (1.18, 1.22)
2017	5024.78 (5023.49, 5026.07)	2.08 (2.05, 2.10)	887.49 (886.95, 888.04)	0.48 (0.47, 0.50)	3822.4 (3821.27, 3823.52)	0.48 (0.46, 0.49)	373.93 (373.57, 374.28)	1.12 (1.10, 1.13)
2018	4953.34 (4952.06, 4954.62)	1.98 (1.96, 2.00)	834.04 (833.51, 834.57)	0.46 (0.44, 0.47)	3821.38 (3820.25, 3822.51)	0.47 (0.46, 0.49)	353.02 (352.68, 353.36)	1.05 (1.03, 1.06)
2019	4914.76 (4913.48, 4916.04)	1.9 (1.87, 1.92)	795.89 (795.38, 796.41)	0.43 (0.42, 0.44)	3835.82 (3834.68, 3836.95)	0.47 (0.46, 0.49)	335.35 (335.02, 335.68)	0.99 (0.98, 1.00)
2020	4921.47 (4920.18, 4922.76)	1.81 (1.79, 1.83)	776.05 (775.53, 776.56)	0.39 (0.38, 0.40)	3875.07 (3873.92, 3876.22)	0.48 (0.46, 0.49)	321.27 (320.95, 321.6)	0.94 (0.93, 0.96)
2021	4935.29 (4933.99, 4936.6)	1.76 (1.74, 1.79)	759.34 (758.83, 759.85)	0.38 (0.36, 0.39)	3916.86 (3915.69, 3918.02)	0.48 (0.47, 0.49)	308.86 (308.53, 309.18)	0.9 (0.89, 0.92)

2022	5024.63 (4592.04, 5457.23)	1.7 (1.53, 1.87)	749.99 (651.19, 848.79)	0.40 (0.30, 0.50)	4007.53 (3828.19, 4186.87)	0.48 (0.37, 0.59)	297.99 (284.57, 311.41)	0.86 (0.82, 0.91)
2023	5059.34 (4217.05, 5901.62)	1.64 (1.33, 1.96)	736.8 (525.8, 947.80)	0.38 (0.20, 0.57)	4063.74 (3738.61, 4388.87)	0.49 (0.24, 0.73)	286.97 (263.50, 310.44)	0.83 (0.74, 0.91)
2024	5100.7 (3740.64, 6460.75)	1.59 (1.09, 2.08)	724.95 (380.25, 1069.64)	0.37 (0.08, 0.66)	4126.14 (3612.44, 4639.85)	0.49 (0.07, 0.91)	276.70 (241.29, 312.10)	0.79 (0.67, 0.92)
2025	5149.25 (3180.29, 7118.21)	1.53 (0.85, 2.22)	714.41 (218.97, 1209.84)	0.36 (-0.04, 0.75)	4196.04 (3457.39, 4934.70)	0.49 (-0.12, 1.11)	267.19 (218.72, 315.66)	0.76 (0.59, 0.93)
2026	5204.74 (2541.86, 7867.62)	1.48 (0.59, 2.37)	705.33 (44.43, 1366.23)	0.34 (-0.17, 0.85)	4273.73 (3274.88, 5272.58)	0.50 (-0.34, 1.34)	258.45 (196.07, 320.82)	0.73 (0.52, 0.95)
2027	5267.13 (1827, 8707.25)	1.43 (0.34, 2.52)	697.57 (-142.12, 1537.25)	0.33 (-0.30, 0.96)	4358.58 (3063.17, 5653.99)	0.50 (-0.59, 1.59)	250.33 (173.34, 327.32)	0.71 (0.44, 0.98)
2028	5336.52 (1036.03, 9637.02)	1.38 (0.08, 2.68)	690.78 (-339.73, 1721.30)	0.32 (-0.43, 1.06)	4450.81 (2822.14, 6079.48)	0.51 (-0.86, 1.87)	242.70 (150.61, 334.80)	0.68 (0.36, 1.01)
2029	5413.34 (167.67, 10659.01)	1.33 (-0.18, 2.84)	684.73 (-547.61, 1917.08)	0.30 (-0.55, 1.16)	4551.89 (2551.41, 6552.36)	0.51 (-1.15, 2.17)	235.55 (128, 343.09)	0.66 (0.29, 1.03)

2030	5497.88 (-780.72, 11776.48)	1.28 (-0.43, 3)	679.36 (-765.22, 2123.94)	0.29 (-0.68, 1.26)	4662.73 (2249.21, 7076.25)	0.52 (-1.47, 2.51)	228.89 (105.58, 352.19)	0.64 (0.22, 1.06)
2031	5589.8 (-1812.78, 12992.38)	1.24 (-0.68, 3.16)	674.86 (-992.54, 2342.26)	0.28 (-0.80, 1.36)	4783.16 (1912.15, 7654.16)	0.53 (-1.81, 2.86)	222.75 (83.36, 362.13)	0.62 (0.14, 1.09)
2032	5689.06 (-2933.02, 14311.15)	1.19 (-0.92, 3.31)	671.25 (-1229.77, 2572.26)	0.27 (-0.92, 1.46)	4912.63 (1535.91, 8289.35)	0.53 (-2.18, 3.25)	217.02 (61.24, 372.81)	0.60 (0.07, 1.12)
2033	5795.78 (-4146.08, 15737.64)	1.15 (-1.16, 3.46)	668.2 (-1476.45, 2812.84)	0.26 (-1.03, 1.55)	5051.79 (1117.61, 8985.96)	0.54 (-2.58, 3.66)	211.61 (39.22, 384)	0.58 (0.01, 1.15)
2034	5910.19 (-5457.38, 17277.75)	1.11 (-1.38, 3.61)	665.51 (-1732.12, 3063.13)	0.25 (-1.14, 1.63)	5201.63 (653.76, 9749.50)	0.55 (-3.01, 4.10)	206.46 (17.32, 395.60)	0.56 (-0.07, 1.19)
2035	6032.44 (-6872.93, 18937.81)	1.07 (-1.6, 3.74)	663.18 (-1996.71, 3323.08)	0.24 (-1.24, 1.72)	5362.50 (139.99, 10585.02)	0.56 (-3.46, 4.58)	201.60 (-4.44, 407.64)	0.54 (-0.13, 1.22)

Abbreviations: STH, soil-transmitted helminth; CI, confidence interval
